# Renal expression of JAK2 is high in polycystic kidney disease and its inhibition reduces cystogenesis

**DOI:** 10.1038/s41598-019-41106-3

**Published:** 2019-03-14

**Authors:** Foteini Patera, Alex Cudzich-Madry, Zhi Huang, Maria Fragiadaki

**Affiliations:** Academic Nephrology Unit, Department of Infection, Immunity and Cardiovascular Disease, University of Sheffield, Sheffield, S10 2RX United Kingdom

## Abstract

Autosomal dominant polycystic kidney disease (ADPKD) is the most common renal genetic disorder, however it still lacks a cure. The discovery of new therapies heavily depends on understanding key signalling pathways that lead to ADPKD. The JAnus Kinase and Signal Transducers and Activators of Transcription (JAK/STAT) pathway is aberrantly activated and contributes to ADPKD pathogenesis via enhancing epithelial proliferation. Yet the mechanisms underlying the upregulation of JAK/STAT activity in this disease context is completely unknown. Here, we investigate the role of JAK2 in ADPKD using a murine model of ADPKD (Pkd1^nl/nl^). In normal kidneys, JAK2 expression is limited to tubular epithelial and vascular cells with lesser staining in bowman’s capsule and remains below detection level in the interstitium. By contrast, in kidneys of mice with ADPKD, JAK2 is higher in cyst-lining cells when compared to normal tubules and critically, it is ectopically expressed in the interstitium, suggesting that ectopic JAK2 may contribute to ADPKD. JAK2 activity was inhibited using either curcumin, a natural compound with strong JAK2 inhibitor activity, or *Tofacitinib*, a clinically used selective JAK small molecule inhibitor. JAK2 inhibition led to significantly reduced tyrosine phosphorylation of STAT3 and markedly reduced cystic growth of human and mouse ADPKD-derived cells in cystogenesis assays. Taken together, our results indicate that blockade of JAK2 shows promise as a novel therapeutic target in ADPKD.

## Introduction

Autosomal dominant polycystic kidney disease (ADPKD) is a devastating multi-organ disease lacking a cure. ADPKD accounts for approximately 5–10% of patients with renal failure. Genetically, it arises predominately due to mutations in the Pkd1 or Pkd2 genes, encoding for the polycystin-1 and polycystin-2 proteins. The disease is characterised by the progressive growth and enlargement of renal cysts. Patients with ADPKD also exhibit a vascular phenotype, presenting with hypertension and aneurysms^1,2^ which together with cysts in kidneys and other organs, shows that ADPKD is a systemic disease. Currently Tolvaptan, a V2-vasopressin receptor antagonist, is the only approved therapy; however, it carries significant side effects^3^ and in is available to some, but not all, patients with ADPKD. Therefore, a major biomedical challenge is to identify central druggable pathways for the treatment of ADPKD.

Curcumin (diferuloylmethane)^4^ is a phytochemical known to exhibit strong JAK/STAT inhibitory activity. Consistent with being a JAK/STAT inhibitor, curcumin exhibits robust antioxidant, anti-tumour and anti-inflammatory effects^5^. Importantly, no appreciable side-effects have been reported making it an attractive drug for long-term inhibition of this pathway. Because of its low toxicity but potency, curcumin has been tested as a therapeutic in ADPKD murine models, where it limites cystic growth^6^ and is currently being trialled in young patients and children with ADPKD (clinicaltrials.gov - NCT02494141).

We have recently shown that JAK2/STAT5 activity is abnormally activated and contributes to ADPKD^7–11^. Others have also shown that additional components of the JAK/STAT pathway are dysregulated in ADPKD, previously reviewed^11^. The mechanisms underlying JAK/STAT hyperactivity in ADPKD are however unknown. STAT transcription factors become activated by a series of phosphorylation events on conserved tyrosine residues facilitated by one of four JAK kinases, namely JAK1-JAK3 or Tyk2. Because of their involvement in disease, JAK small-molecule inhibitors have been developed, including the approved Tofacitinib, Ruxolitinib, and Baracitinib^12–15^. Moreover, additional JAK inhibitors are in clinical trials, making JAK-STAT a therapeutically tractable pathway. Evidence exists that STAT1, STAT3, STAT5 and STAT6 are involved in ADPKD pathogenesis (^7–9,11,16–23^), while on the contrary the involvement of JAK kinases, which are druggable, has not been previously studied in ADPKD. Combining our previous and current work with that of other groups we predict that JAK-STAT inhibition may be of therapeutic benefit in ADPKD. We therefore investigated the expression of JAK2 in kidneys of mice with and without ADPKD and inhibited JAK2 activity to examine its role in cystogenesis.

## Results

### JAK2 is highly expressed by both cystic and normal renal epithelial cells *in vivo*

**Figure 1:**
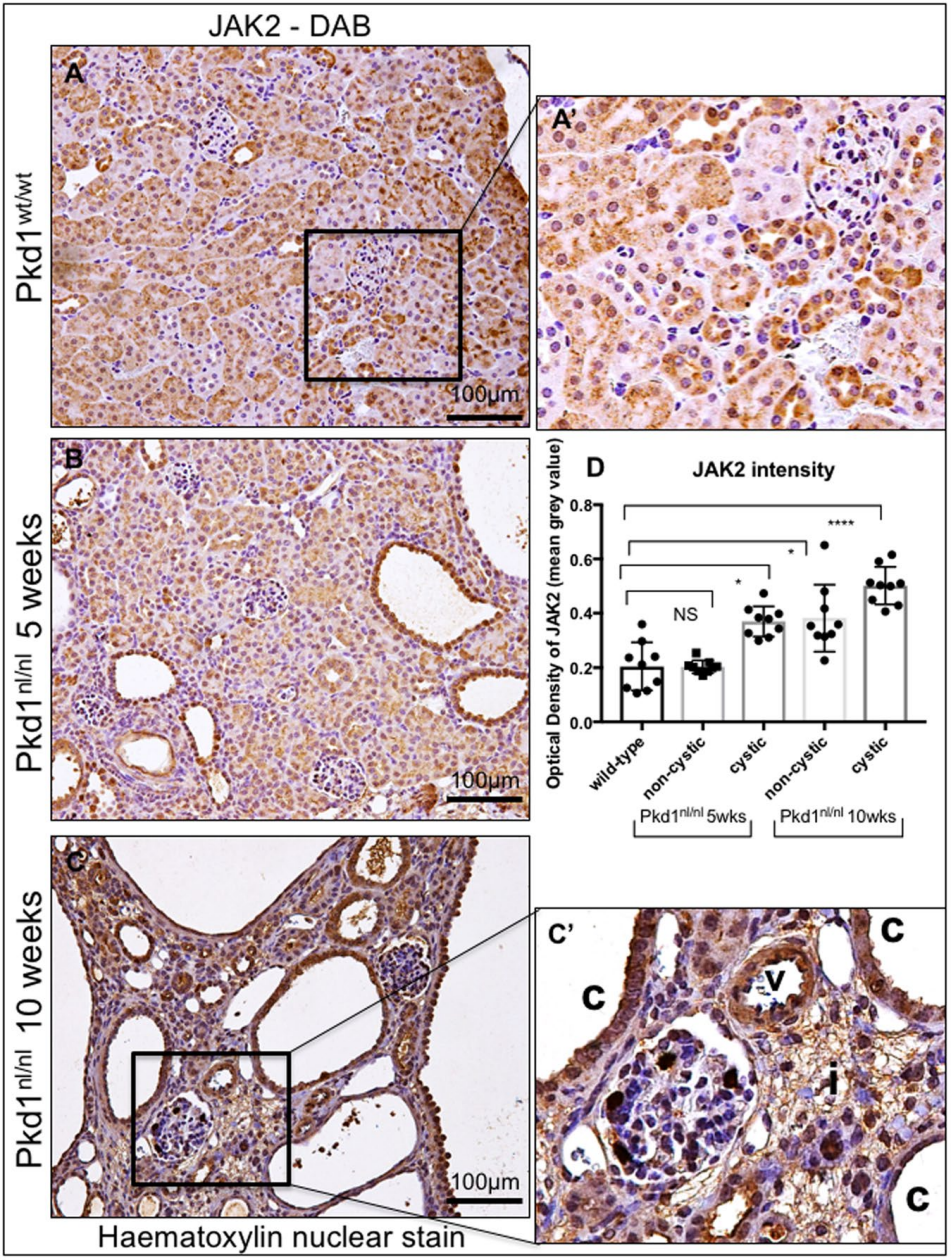
JAK2 is highly expressed by cystic renal epithelial cells *in vivo*. JAK2 levels were studied in kidneys sections of the Pkd1^nl/nl^ model at 5 and 10 weeks of age (5 mice in each group), representative images at 20x magnification are shown. Kidneys of Pkd1 wild type littermates exhibit JAK2 staining through the kidney parenchyma with strong expression in tubules (**A**). 5 weeks of age Pkd1^nl/nl^ mice exhibit JAK2 expression in both cystic and non-cystic tubules (c, denotes cysts) (**B**). While at 10 weeks of age the cysts of the Pkd1^nl/nl^ are larger and JAK2 is highly expressed in epithelial (**C** and C’) but also ectopically expressed in the interstitium (i). Expression of JAK2 is also found in vessels (v). JAK2 quantification revealed stronger expression in cystic epithelial when compared with non-cystic and with wild-type normal tubules (**D**).

To investigate whether JAK2 is involved in the development of cysts *in vivo*, we stained kidney sections from the Pkd1^nl/nl^ mouse model^24^ and associated wild-type littermate controls with anti-JAK2 antibodies. Kidneys from 5 weeks old mice, with intermediate disease, and 10 weeks old with advance disease, were studied as previously described^7^. We found that JAK2 is diffusely expressed by cortical renal epithelial cells and its expression appears predominately cytoplasmic with no detectable expression by fibroblasts or the interstitium (Fig. 1A and A’). During early stages of ADPKD, JAK2 is strongly expressed by renal cystic tubules, and to a lesser extent by non-cystic, otherwise normal, tubular epithelial cells (Fig. 1B). At 10 weeks of age, as the disease advances evident by increased number of larger cysts and diffuse interstitial expansion, JAK2 is strongly expressed in cystic epithelial cells and this expression appears to be stronger in cystic than in healthy non-cystic tubules. Quantification of JAK2 staining intensity indicated that mice with intermediate and late stages of disease (5 and 10 weeks) have stronger JAK2 in cystic when compared to non-cystic tubules and compared to wild-type littermate animals (Fig. 1D). Some JAK2 expression is detected in interstitial cells in mice with ADPKD (Fig. 1C’) which is absent in healthy kidneys (Fig. 1A’), suggesting that JAK2 expression in the interstitium is ectopic. JAK2 is expressed in vessels with a lesser expression by bowman’s capsule cells (Fig. 1C’). Expression of JAK2 in vessels remains strong as disease progresses (Fig. 1C). Hence, JAK2 expression is temporally and spatially coincident with cyst growth in the Pkd1^nl/nl^ mouse model of ADPKD.

### STAT3 tyrosine phosphorylation is potently suppressed by curcumin

**Figure 2:**
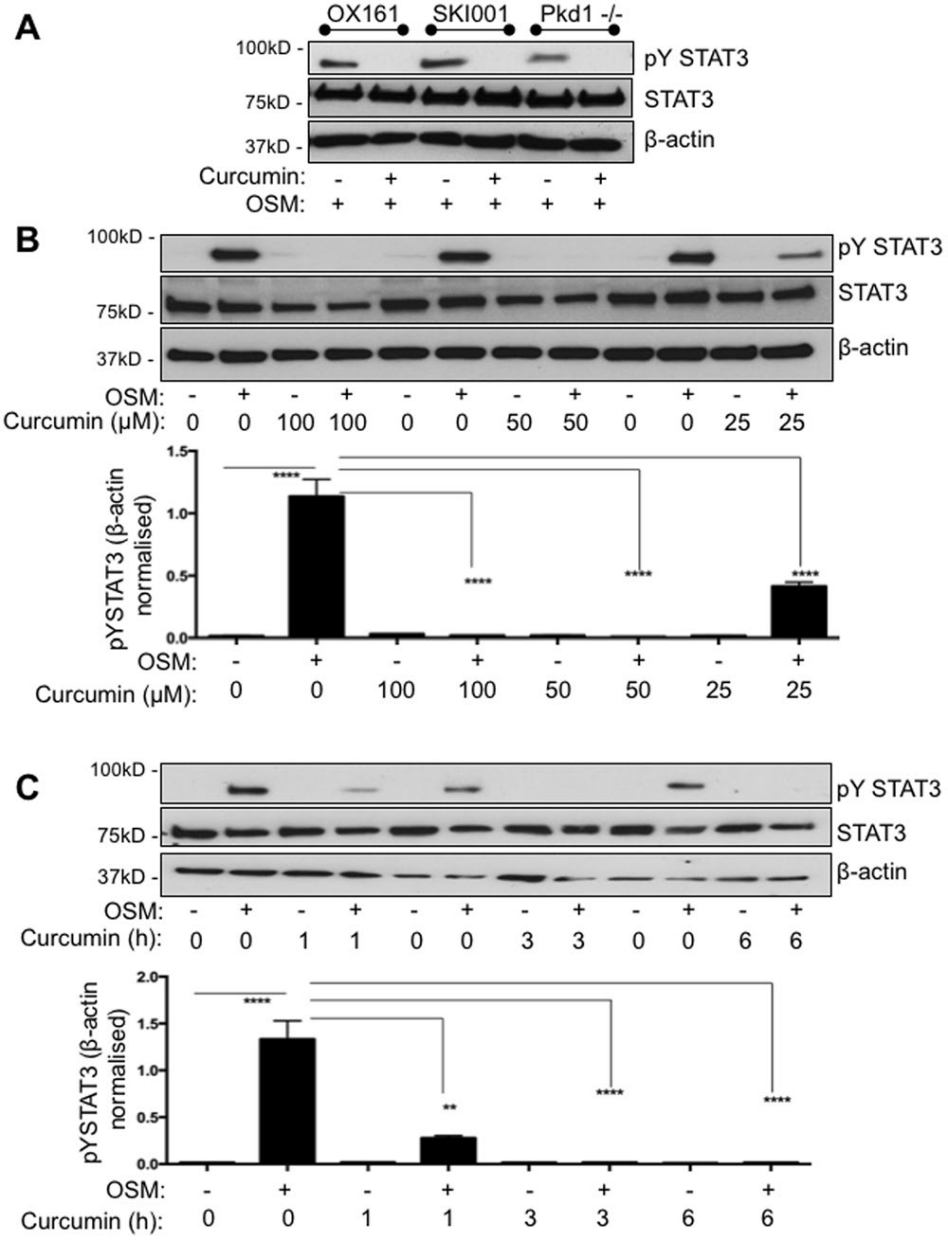
Curcumin is a potent STAT3 inhibitor in renal ADPKD-derived epithelial cells. (**A**) Oncostatin M (OSM, 10 ng/ml) was used to stimulate phosphorylation of STAT3. Western blotting was performed with total cell lysates from human ADPKD-derived epithelial cells OX161-c1, SKI-001 and mouse F1 Pkd1^−/−^. Anti- STAT3 phospho-tyrosine 705 antibody (pY STAT3), total STAT3 (STAT3) and β-actin, which served as an internal loading control, were used. In all three cell lines curcumin treatment inhibited OSM-induced pY STAT3. (**B**) SKI001 cells treated with either 100, 50 or 25 μM of curcumin were subjected to blotting for STAT3, pYSTAT3 and β-actin control. Densitometric quantification of three independent blots was carried out. One-way Anova with Bonferroni corrections was performed, and values lower that 0.05 were considered statistically significant. (**C**) SKI001 cells were treated with 100 μM of curcumin for 1 or 3 or 6 hours and stimulated either with water vehicle control or 10 ng/ml of OSM. pYSTAT3, STAT3 and β-actin were investigated by blotting. Quantification of three independent blots was carried out. One-way Anova with Bonferroni corrections was performed, and values lower that 0.05 were considered statistically significant. Symbol meaning: *P ≤ 0.05, **P ≤ 0.01, ***P ≤ 0.001, ****P ≤ 0.0001.

Since JAK2 is highly expressed in cystic epithelial cells *in vivo* (Fig. 1) we considered whether blocking JAK2 activity may offer protection against cystogenesis and set out to explore this biochemically *in vitro*. Studies in non-renal systems, with intact Pkd1-triggered signalling, have suggested that curcumin acts as a strong JAK-STAT inhibitor^25–27^, an observation confirmed by our studies (Supplementry Fig. 1A). We used curcumin to inhibit JAK/STAT activity in three independent ADPKD-derived epithelial cell lines; three cell lines were used to avoid cell-line specific artefacts. All ADPKD lines tested here did not show signs of constitutive activation of JAK/STAT, hence to activate the pathway, we used a defined amount of oncostatin M (OSM)^28^. 50μΜ of curcumin for 6 hours potently inhibited the OSM-induced STAT3 activity, indicated by a marked reduction in the level of tyrosine phosphorylation (Fig. 2A), without affecting total STAT3 protein levels. We next performed a time-response curve, which showed that one-hour of curcumin treatment is sufficient to cause nearly 50% reduction in STAT3 phosphorylation while 3 and 6 hours result in 100% reduction. Total STAT3 protein levels were unaltered by the treatment (Fig. 2B). To study the amount of curcumin needed to cause 50% decrease in phosphorylation of STAT3, cells were stimulated for three hours with 100, 50 or 25 μM of curcumin. Treatment with 100 μM and 50 μM caused 100% reduction in tyrosine phosphorylation, while 25 μM only reduced it by 50%. These time- and dose-dependent data show that curcumin potently blocks STAT3 activity in ADPKD-derived epithelial lines.

### Curcumin blocks STAT3 activity via JAK2

**Figure 3:**
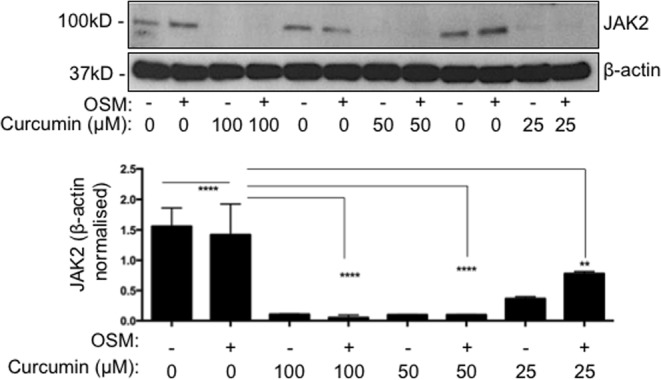
Curcumin inhibits JAK2. SKI001 cells were exposed to 100 or 50 or 25 μM of curcumin and immunoblot was carried out using anti-JAK2 and β-actin antibodies. Total JAK2 is found in both OSM stimulated and unstimulated cells. Quantification of three independent immunoblots was carried out. One-way Anova with Bonferroni corrections was carried out and values lower that 0.05 were considered statistically significant. Symbol meaning: *P ≤ 0.05, **P ≤ 0.01, ***P ≤ 0.001, ****P ≤ 0.0001.

To find out how curcumin blocks STAT3 activity we studied JAK2, which is highly expressed by cyst-lining cells *in vivo* (Fig. 1) and is known to activate STAT3 by phosphorylation. We found that 100 μM and 50 μM of curcumin caused approximately 100% reduction in JAK2 levels, while 25μΜ resulted in a modest 50% decrease (Fig. 3). Curcumin reduced JAK2 levels in both OSM-treated and control-treated cells, hence showing that it affects JAK2 in an OSM-independent manner. Curcumin’s effect on JAK2 activity was consistent with the blockade of STAT3 phosphorylation observed above (Fig. 2). To test if this effect was not cell line specific, we tested the effect of curcumin in mouse F1-Pkd1^−/−^ cells and found that curcumin reduced JAK2 levels (Supplementry Fig. 2B). Therefore, curcumin blocks JAK2 and this may explain the reduced STAT3 activity.

### Curcumin does not change JAK2 levels but makes JAK2 insoluble

**Figure 4:**
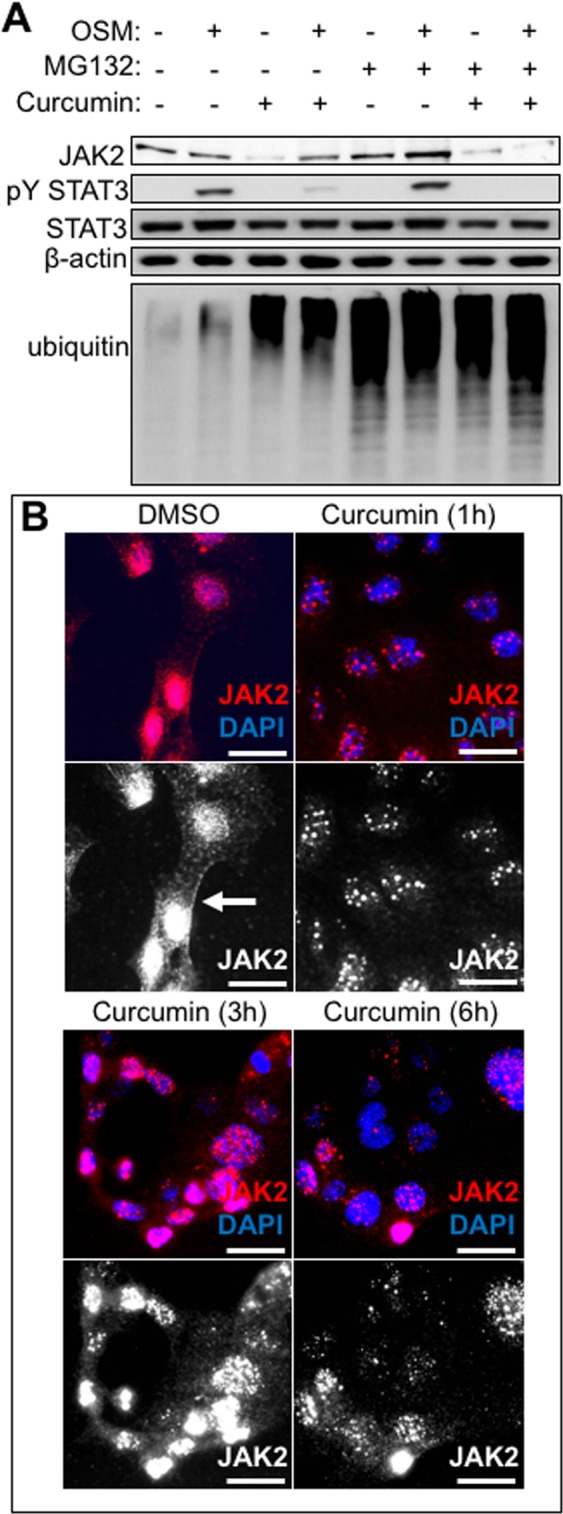
Curcumin controls JAK2 localisation. (**A**) SKI001 cells were treated with OSM (10 ng/ml), or MG132 (50 μM) or curcumin (50 μM) for 3 hours and lysates were subjected to immunoblotting. Antibodies against JAK2 pYSTAT3, STAT3, ubiquitin (indicator of proteasome inhibition control) and β-actin (internal loading control) were studied. (**B**) SKI001 cells were either treated with DMSO vehicle control for 3 hours (3 h) or curcumin for 1 h or 3 h or 6 h, they were then snap frozen on ice-cold methanol, stained with anti-JAK2 rabbit antibody, followed by an anti-rabbit AF594 and imaged in an inverted fluorescent microscope. Red is anti-JAK2 (also in greyscale in lower panel), blue is nuclear counterstain (DAPI). Scale bars are 25 μm.

We next wished to understand how curcumin causes the apparent alteration of the levels of JAK2 in immunoblots. Firstly, we explored the option that curcumin may cause JAK2 degradation via proteasomal processing of JAK2. To assess this, we treated cells with MG132, a proteasome inhibitor. MG132 caused the expected increase in ubiquitinated proteins, thus confirming adequate level of proteasomal blockade (Fig. 4A). Yet, the effect of curcumin on JAK2 did not depend on the proteosome, as MG132 was unable to restore JAK2 levels following curcumin treatment (Fig. 4A compare lane 3 with 7 and 8). These data show that curcumin-induced JAK2 reduction is not though the proteasome. Given that the proteasome is not responsible for the decreased JAK2 levels we decided to perform immunocytochemistry to visualise JAK2 and test whether curcumin treatment affects its subcellular localisation. Interestingly, we found that JAK2 is found throughout the cell body with some localisation in the plasma membrane in DMSO-vehicle treated cells, however curcumin treatment causes JAK2 to become strongly punctate (Fig. 4B). Previous studies have suggested that curcumin can cause protein precipitation into aggresomes^29,30^, we therefore suggest that these punctate structures may be JAK2 aggregates. This could explain how JAK2 becomes insoluble and provide an explanation as to why we were unable to detect JAK2 in the detergent-soluble fraction by immunoblotting. Taken together, our data show that curcumin causes JAK2 inactivation without involving the proteasome.

### Curcumin-induced JAK2 blockade reduces cystic growth *in vitro*

**Figure 5:**
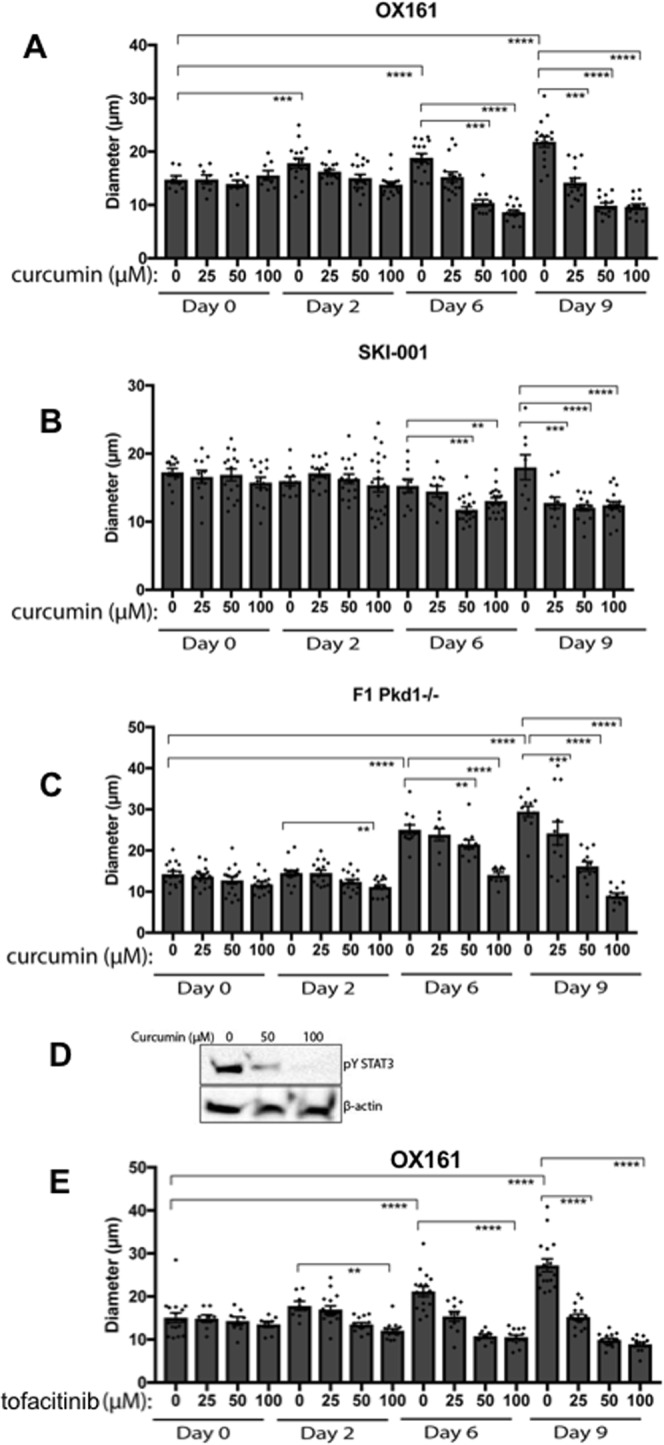
Curcumin slows down cyst growth *in vitro*. ADPKD-derived human epithelial cells were grown in BD-Matrigel to allow the formation of cysts to grow over time. All cysts formed were photographed at day 0, 2, 6 and 9; using an Olympus inverted microscope. Curcumin at 100, 50, 25 and 10 μM or DMSO vehicle-control was added and cyst growth measured in (**A**) human OX161 (**B**) human SKI-001 and (**C**) mouse derived Pkd1^−/−^ cell lines. (**D**) Immunoblots were performed from cell lysates obtained from cells grown in cysts at day 9. Matrigel was melted and pYSTAT3 was measured, β-actin was also assessed. Cells were treated with either 0, 50 or 100 μM of curcumin to test whether the latter can reduce JAK/STAT activity in the cysts. One-way Anova with Bonferroni corrections was carried out and values lower that 0.05 were considered statistically significant. Symbol meaning: *P ≤ 0.05, **P ≤ 0.01, ***P ≤ 0.001, ****P ≤ 0.0001.

To study the ability of the JAK2/STAT3 pathway to drive cystogenesis we performed cystogenesis assays *in vitro* using a monoculture grown in three dimensions (3D cyst assays). We used three independent cell lines to ensure the robustness of results and avoid cell-type specific artefacts. In these assays microscopic cysts formed, from each independent cell line, which grew in diameter over time, as previously shown by our group^7^. We found that curcumin-induced JAK2 blockade led to a clear and reliable reduction in the size of cysts in all three independent cell lines tested and, in a time and dose-dependent manner (Fig. 5). Critically, two of the three cell lines used are human derived cells, therefore highlighting human relevance. Curcumin reduced the growth of cysts at day 2, with the effect persisting to day 6 and 9. Curcumin at 50 and 25μΜ also clearly reduced cyst size at day 9 in OX161-c1 cells, which are human-derived ADPKD cells (Fig. 5A). A similar pattern was seen in SKI-001, an additional independent human ADPKD-derived line, which showed reduced cyst size at day 6 and 9 with 50 and 25μΜ of curcumin (Fig. 5B). We also studied a mouse line which has a Pkd1 deletion and is therefore considered as a mouse ADPKD line, as expected curcumin blocked the growth of cysts at day 2 with 100 μM of curcumin, while day 6 and day 9 50 and 100μΜ were able to cause a block in the growth of cysts (Fig. 5C). Pkd1 wild-type F1 mouse renal cells also responded by exhibiting reduced cystic growth (data not shown), suggesting that the strong anti-cystogenic effect of curcumin is Pkd1-independent. To study whether JAK/STAT in indeed activated in cells grown in 3D cyst assay and investigate if curcumin is able to block STAT3 phosphorylation, we performed immunoblotting from cyst assays. STAT3 is phosphorylated within the cysts and curcumin potently blocks this (Fig. 5D), as well as reducing JAK2 levels (Supplementry Fig. 1C). These data suggest that the previously reported anti-cystic actions of curcumin may be via blockade of JAK/STAT. Given that curcumin is pleiotropic and can block pathways other than just JAK/STAT we also used a selective JAK small-molecule inhibitor, namely tofacitinib, which is currently in clinical use for rheumatoid arthritis. We found that tofacitinib led to a significant reduction in cyst size (Fig. 4E), thus suggesting that JAK inhibition alone is sufficient to block cystic growth of ADPKD cells.

## Discussion

We show that JAK2 levels are higher in polycystic when compared with wild-type kidneys a finding that may be therapeutically relevant given that we identified that JAK blockade, using either curcumin or a selective JAK inhibitor, potently reduces cystic growth in human ADPKD-derived lines. These data are important because multiple components of the JAK/STAT pathway contribute to cystogenesis, and JAK activity is therapeutically amenable, therefore providing a realistic potential for intervention studies. Our data suggest that JAK2 is a key kinase promoting cystic growth in ADPKD, since inhibiting it, blocks cystic growth of ADPKD cells. JAK2 may contribute to the development of cystic growth likely via canonical activation of one, or multiple, of the downstream STAT transcription factors. Indeed, a number of groups, including our own, have shown that STAT3, STAT5 and STAT6, which are normally latent transcription factors, are activated in ADPKD^7,10,11,18–21,23,31^. JAK2 is involved in tyrosine phosphorylation of all of these STATs^32–34^, which is an essential step for the dimerization and activity of these transcription factors. Therefore, the inhibition of cystic growth observed under JAK blockade may be via suppression of canonical JAK/STAT activity. Alternately, STAT transcription factors can also be activated, non-canonically, by other kinases such as Src, which was found to be responsible for activating STAT3 in MDCKII renal cells^31^. Indeed, curcumin can the biochemical activity of the Src kinase^35^ in addition to blocking JAK/STAT activity. If STATs are activated by Src or other kinases, then it follows that highlt selective small-molecule inhibitors of JAKs would not be effective in reducing JAK/STAT activity and lowering cystic growth. However, our work shows that tofacitinib, which is a selective JAK inhibitor, used at concentrations found in plasma of treated individuals, resulted in reduced cyst growth. Therefore, we conclude that JAK2 is a major kinase driving cystogenesis of ADPKD cells.

The choice of JAK inhibitor is based on a number of considerations, including how effective a given drug is and balancing this with its side effects. Over the years, phytochemicals, such as curcumin, have been used to inhibit, prevent or reverse disease processes. Curcumin for example has strong anti-cystic effects in pre-clinical models of ADPKD^6^. One advantage of curcumin is that it can be used long term, due to lack of apparent side effects^36^. However, the bioavailability of curcumin is low^37,38^, making it less attractive when compared with small molecule inhibitors, such as tofacitinib, the JAK inhibitor used in our study. Indeed, here we showed that both curcumin and tofacitinib were capable of slowing down the rate of growth of cysts in cystogenesis assays. A comparison of tofacitinib (and other JAK inhibitors) with curcumin in a pre-clinical setting is required to assess effectiveness and measure their side effects in murine models of ADPKD.

Previous studies used systemic administration of curcumin in a murine model of ADPKD^6^, where curcumin showed promising results; however, whether curcumin acted primarily on renal epithelial or vascular cells was uncertain from these studies. Here, we find that JAK2 is expressed in both vascular and epithelial cells in the ADPKD affected kidney. To gain a deeper understanding we used monoculture assays and found that curcumin reduces cystogenesis of renal epithelial cells without requiring interactions with vascular cells. As such, if, and how, curcumin affects vessels is currently unknown. However, curcumin is currently under clinical investigation (phase 4 clinical trial) for its potential to modify the vascular phenotype in children and young individuals with ADPKD. The results of this study may determine whether curcumin can protect the vasculature of young individuals affected with against ADPKD, which will provide an additional protective mechanism for this drug.

Curcumin causes JAK2 to move into an insoluble fraction, and we observe the generation of large JAK2 positive puncta in cells treated with curcumin. We do not know the nature of these puncta, however, curcumin has been previously shown to cause protein aggregation^29^, therefore we propose that these structures may be aggresomes. The observation that these JAK2 positive puncta are not dependent on proteasomal processing of JAK2 is evidence that JAK2 has not been degraded. Likewise, others have reported that curcumin-treated cancer cells respond by a temporal blockade of STAT3, which is fully reversible within 24 hours^39^, this is consistent with our observations. Therefore, we propose a model whereby curcumin takes JAK2 briefly out of action by causing it to aggregate, without leading to JAK2 degradation, which is an irreversible action. It should be noted that the effects of tofacitinib are also reversible but it requires up to 14 days for reversibility. This is important because it may explain the lack of significant side effects in response to systemic treatment with curcumin.

In summary, we show that JAK2 is highly expressed in polycystic kidneys and its blockade reduces cystic growth. Understanding how key signalling pathways, such as the JAK/STAT, are misregulated in ADPKD will help identify suitable molecules to target for therapy. Based on our *in vivo* immunohistochemical analysis an *in vitro* cyst assays in human cells, we propose that JAK inhibitors may be of therapeutic benefit for patients with ADPKD. More studies are required to explore the mechanisms employed by JAK-STAT to regulate cystic growth and to identify the most suitable point in the pathway to intervene. In conclusion, our study provides the basis for further testing of JAK inhibitors as a novel therapeutic avenue for patients with polycystic kidney disease.

## Methods

### Cell lines

Conditionally-immortalized ADPKD-derived lines (OX161-c1 and SKI-001)^40^ are tubular epithelial cells isolated from human kidneys and immortalized by transduction at an early passage (P1-4) with a retroviral vector containing a temperature-sensitive large T antigen and the catalytic subunit of human telomerase^41^. The F1-Pkd1 WT renal epithelial cells were previously isolated from kidney papillae of the Pkd1fl/fl mouse (B6.129S4-Pkd1tm2Ggg/J; the Jackson Laboratory) and were immortalized with the lentiviral vector VVPW/mTert expressing the mTert; to delete Pkd1 and produce F1/Pkd1−/− cells they were subsequently transfected with VIRHD/HY/Silntβ1/2363 lentivectors followed by hydromycin selection^42^.

### Cyst assays

Cyst assays were performed as previously described^7^. In brief, for ADPKD cystic cell lines, 2 human lines namely OX161-c1, SKI-001, and one mouse line F1-Pkd1^−/−^ were used. OX161 and SKI-001 were kept at 33 °C F1-Pkd1−/− were at 37 °C. Healthily growing cells were trypsinised and re-suspended in DMEM media containing 10% FBS (no antibiotics) and counted using a haemocytometer. Subsequently 2 × 10^4^ cells per well were mixed with basement Matrigel (354230, BD biosciences). Prior to use, the Matrigel was left to thaw on ice and once cells were added it was immediately loaded onto the 96 well plate, allowing it to polymerise. 100 μl of DMEM media supplemented with 10% FBS was added to each well and cells returned to the incubator for 24 hours to allow them to recover. After 24 hours, the media was aspirated and replaced with fresh media (DMEM + 10% FBS) supplemented with either vehicle (DMSO) or curcumin (Sigma). The cells were photographed before every media change and every two days, the media was replaced with fresh media containing either curcumin or DMSO.

### Immunohistochemistry/immunofluorescence

Cells grown on coverslips were fixed with ice-cold methanol prior to blocking in 2% milk/TBST for 30 minutes and incubated with primary antibodies overnight at 4 °C. Antibody used was anti-rabbit JAK2 (3230S, cell Signalling, D2E12). Cells were washed and incubated with secondary antibody, anti-rabbit AF594 (A-11037, Invitrogen (1:250). Microscopy was carried out using a confocal microscope. Immunohistochemistry using a JAK2 antibody (3230S, Cell Signalling) was performed as previously described^7^. Quantification of JAK2 staining intensity was performed using Fiji freeware. Image was first colour-convoluted using the H -DAB function, image 2 (DAB) was converted to 8-bit and using the freehand selection tool, an area of interest was manually drawn around each tubule, mean grey intensity, maximum intensity (which is 255 for an 8-bit image) were obtained for each measured tubule. Data are presented as optical density (mean grey value) and were calculated following the formula Optical density = log(max intensity/mean intensity). To minimize the biases, which can be introduced in areas where there is no tissue (middle of a cyst), the non-cell compartment was excluded by drawing around the cysts.

### Western blotting and curcumin treatment

Cells were analysed by Western blotting with previously reported protocols^43–46^. In brief, cells were lysed using ice-cold Lysis Buffer (50 mM Tris (pH 7.4) 250 mM NaCl, 0.3% Triton X-100, 1 mM EDTA) supplemented with protease inhibitor cocktail (Roche), freeze-thawed and sonicated. Whole cell lysates were boiled in 2xLaemmeli sample buffer for 5 minutes. Samples were resolved by SDS-PAGE and transferred using the Mini-PROTEAN system (Bio-Rad). Primary antibodies were anti-β-actin (ab8226, Abcam), anti-phosphorylated STAT3 (p-STAT3) (9145, Cell Signalling), anti-STAT3 (12640, Cell Signalling). Curcumin (08511-10MG, Sigma) was resuspended in DMSO and used at stated doses, DMSO served as vehicle control. For immunoblotting in cyst assays, Matrigel was first dissolved using cultrex organoid harvesting solution, then cysts were lysed directly in 2xLaemmeli sample buffer and immunoblots carried out as described above.

### Animals

*Pkd1*^nl/nl^, harboring an intronic neomycin-selectable marker, or wild type littermate controls were used^7,47^. *Pkd1*^nl/nl^ or control mice were sacrificed at 5 or 10 weeks of age and kidneys collected, formalin-fixed and paraffin embedded. 5-micron section were used for histological examination. All mouse experiments were done under the authority of a U.K. Home Office license (license holder Dr Maria Fragiadaki, license number PPL7008968).

### Statistical analysis

Data were analysed using Prism GraphPad and Non-parametric, two-tailed, Mann-Whitney T-tests or one-way ANOVA were performed. Results with a P value of 0.05 or lower were considered statistically significant^24^.

## Supplementary information

Sup Figure 1

## Data Availability

All data including supporting datasets are made available as main figures or supplementary information files.
