# Supplementary material for: Renal expression of JAK2 is high in polycystic kidney disease and its inhibition reduces cystogenesis
Source: Sci Rep. 2019 Mar 14;9:4491. doi: 10.1038/s41598-019-41106-3 (PMC6418191; doi:10.1038/s41598-019-41106-3)

Renal expression of JAK2 is high in polycystic kidney disease and its inhibition reduces cystogenesis.

Foteini Patera[1], Alex Cudzich-Madry[1], Zhi Huang[1] & Maria Fragiadaki[1]*

Sup Figure 1

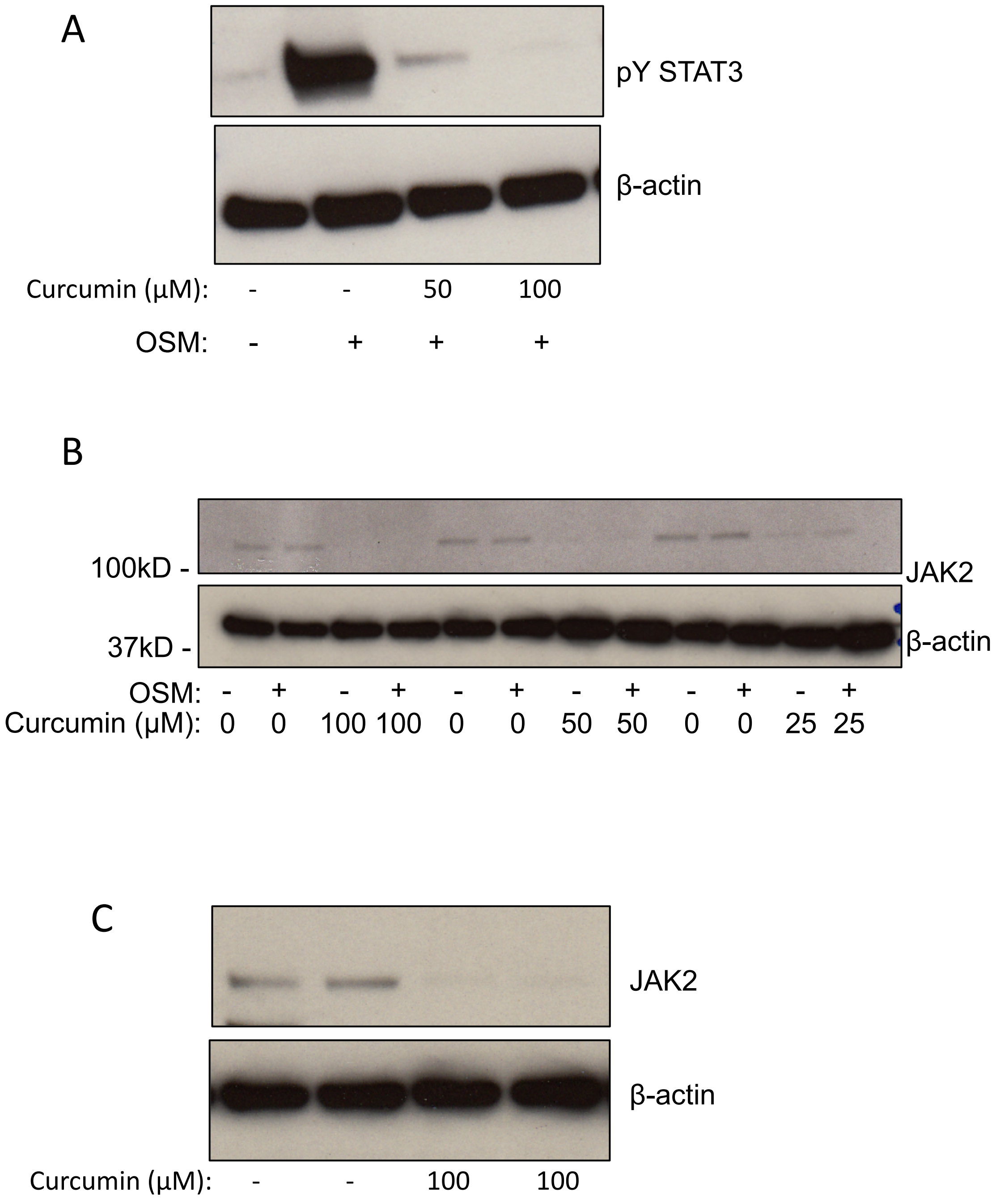

Sup Figure 1: **A.** PYSTAT3 was immunoblotted in Pkd1 WT renal tubular epithelial cells. Curcumin reduces STAT3 activity irrespectively of Pkd1 status **B.** JAK2 immunoblot in Pkd1-/- cells, treated either OSM to induce JAK/STAT activity, or left untreated and treated with curcumin at defined concentrations, as indicated. **C.** JAK2 immunoblot from cyst assays, showing that JAK2 is active in the cystogenesis assays and curcumin inhibits JAK2.

Unmodified western blots

Figure 2A

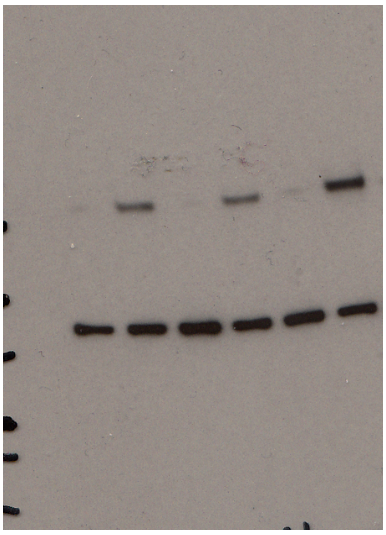

Figure 2B

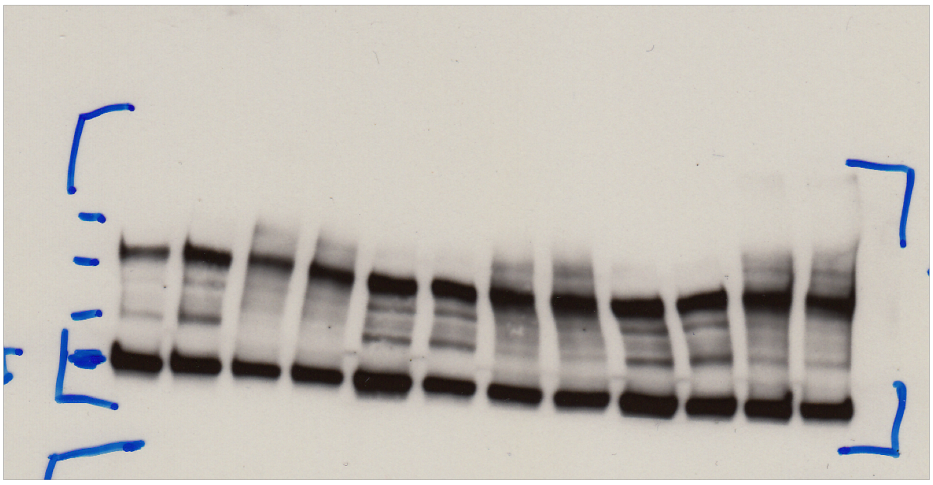

Figure 2C

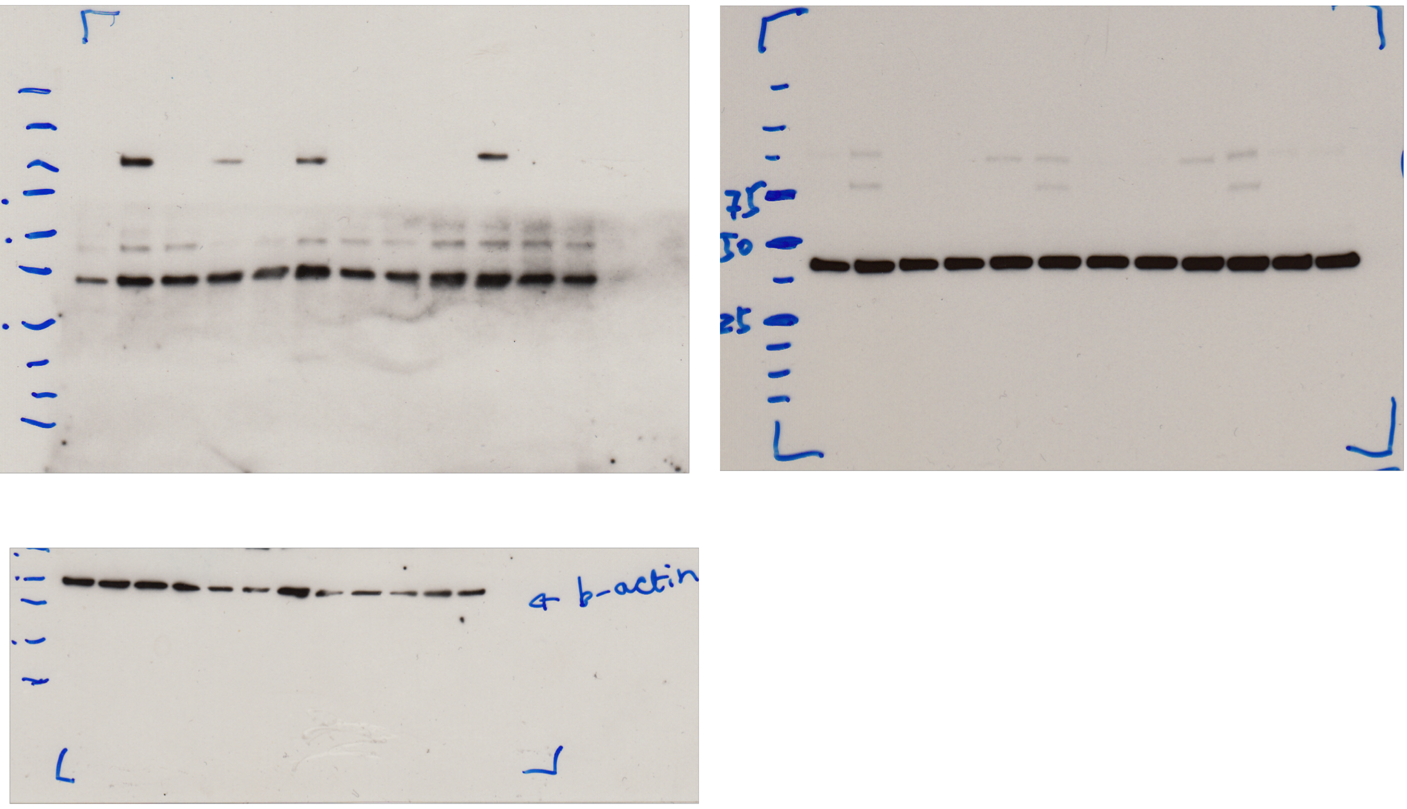

Figure 3

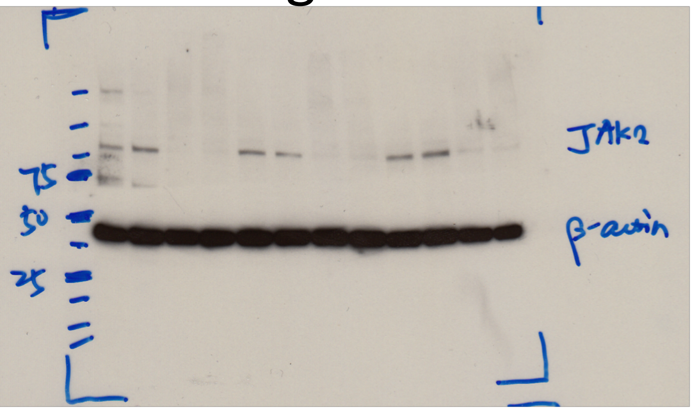

Unmodified western blots

Figure 4

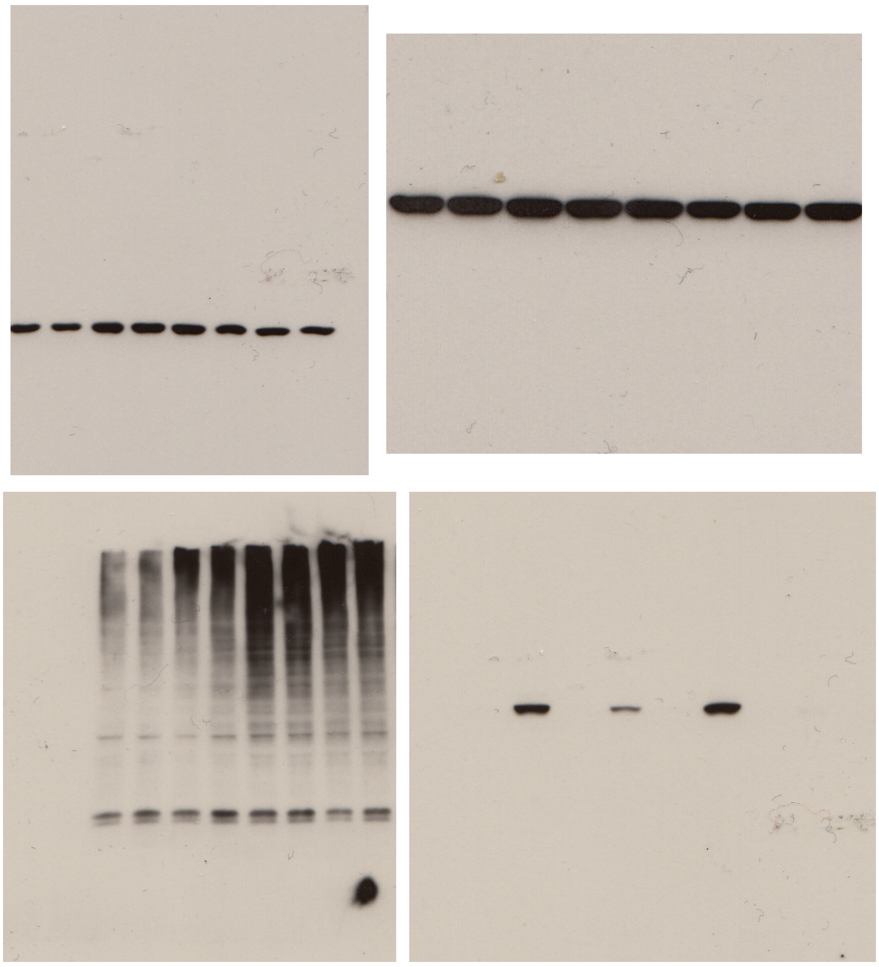

FIGURE 5

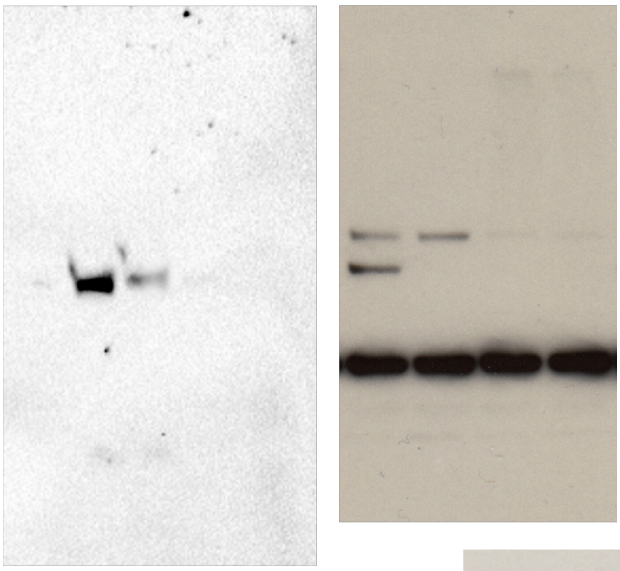

Sup Fig 1

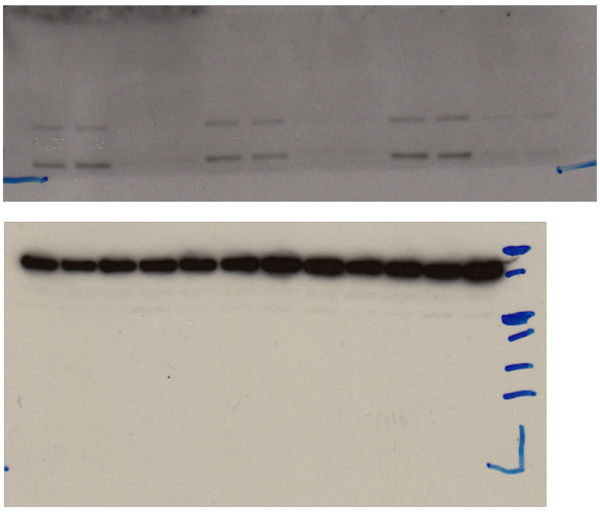

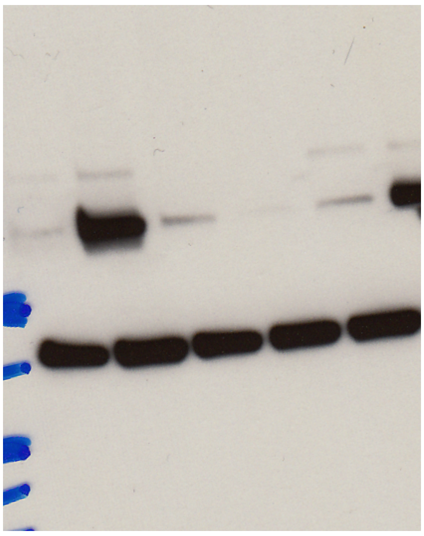

Supplement: Supplementary file 1 — Sup Figure 1 [file 41598_2019_41106_MOESM1_ESM.pdf]
